# Detection of clusters of a rare disease over a large territory: performance of cluster detection methods

**DOI:** 10.1186/1476-072X-10-53

**Published:** 2011-10-04

**Authors:** Stéphanie Goujon-Bellec, Claire Demoury, Aurélie Guyot-Goubin, Denis Hémon, Jacqueline Clavel

**Affiliations:** 1 INSERM, CESP Environmental epidemiology of cancer, U1018, Villejuif, France; 2 University Paris-Sud 11, UMRS-1018, Villejuif, France; 3 French National Registry of Childhood Hematological malignancies (NRCH), Villejuif, France

**Keywords:** Power, Cluster detection, Rare disease, Leukemia, Large scale, Spatial scan methods

## Abstract

**Background:**

For many years, the detection of clusters has been of great public health interest. Several detection methods have been developed, the most famous of which is the circular scan method. The present study, which was conducted in the context of a rare disease distributed over a large territory (7675 cases registered over 17 years and located in 1895 units), aimed to evaluate the performance of several of the methods in realistic hot-spot cluster situations.

**Methods:**

All the methods considered aim to identify the most likely cluster area, i.e. the zone that maximizes the likelihood ratio function, among a set of cluster candidates. The circular and elliptic scan methods were developed to detect regularly shaped clusters. Four other methods that focus on irregularly shaped clusters were also considered (the flexible scan method, the genetic algorithm method, and the double connected and maximum linkage spatial scan methods). The power of the methods was evaluated via Monte Carlo simulations under 27 alternative scenarios that corresponded to three cluster population sizes (20, 45 and 115 expected cases), three cluster shapes (linear, U-shaped and compact) and three relative risk values (1.5, 2.0 and 3.0).

**Results:**

Three situations emerged from this power study. All the methods failed to detect the smallest clusters with a relative risk lower than 3.0. The power to detect the largest cluster with relative risk of 1.5 was markedly better for all methods, but, at most, half of the true cluster was captured. For other clusters, either large or with the highest relative risk, the standard elliptic scan method appeared to be the best method to detect linear clusters, while the flexible scan method localized the U-shaped clusters more precisely than other methods. Large compact clusters were detected well by all methods, with better results for the circular and elliptic scan methods.

**Conclusions:**

The elliptic scan method and flexible scan method seemed the most able to detect clusters of a rare disease in a large territory. However, the probability of detecting small clusters with relative risk lower than 3.0 remained low with all the methods tested.

## Background

For many years, the detection of clusters has been of great public health interest and widely studied. Several hypotheses may explain the finding of spatial clusters, of which the presence of environmental risk factors, possibly localized in space and time. Several methods have been developed to detect clusters and their performance has been evaluated in various contexts. The most famous method, the circular scan method [1], is generally considered the gold standard. The method is advantageous in that it is easy to use thanks to the freely available SaTScan software. This method is, however, known to be less capable of precisely detecting non-circular clusters. In recent years, several methods have been developed to detect arbitrarily shaped clusters. Based on a moving window of varying size, the elliptic scan method [2] enables detection of circular and elliptic clusters. The flexible scan method is free from the regular shape constraints and considers all the connected zones included in a given neighborhood of each geographic unit as cluster candidates [3]. The method may be computer intensive. Other methods use graph-based algorithms that rely on the geographic neighbor structure of the territory instead of the geographic coordinates of the unit centers ([4-6] and Costa MA, Assunção RM, Kulldorff M: Constrained spanning tree algorithms for irregularly shaped spatial clustering, submitted). The upper level set scan statistic developed by Patil and Taillie [6] is a special case of the static minimum spanning tree method, which has been shown to be far less powerful than the dynamic spanning tree method (dMST) [4]. However, the dMST method tends to detect wide clusters with long branches similar to tentacles, a phenomenon referred to as the 'octopus effect' in the literature. Three constrained spanning tree methods, the early-stopping dMST, the double connected method and the maximum linkage method, were developed by Costa et al. to resolve the problem (Costa MA, Assunção RM, Kulldorff M: Constrained spanning tree algorithms for irregularly shaped spatial clustering, submitted). Among the methods, which are closely related to the dMST approach, the early-stopping dMST appeared significantly less powerful with regard to detecting clusters. Duczmal's simulated annealing method [5], also based on the geographic graph structure, selects the most promising connected zones of any shape over the whole territory as cluster candidates. The genetic algorithm more recently developed by the same team appeared far less time consuming than, and as powerful as, the simulated annealing method for detection of the presence of particular circular and irregularly shaped clusters [7]. To deal with the 'octopus effect' problem, Duczmal et al. considered a non-compactness penalty function defined so as to penalize irregularly shaped cluster candidates [5,7].

Until now, the power of cluster detection methods has mainly been evaluated by comparison with the performance of the circular or elliptic scan methods, but three or more of those methods have rarely been compared. Moreover, the great heterogeneity of the literature, particularly with regard to study design and the evaluation metrics under consideration, renders between-published study comparisons difficult. In addition, the methods were mostly evaluated on a territory of more limited extent in terms of the number of geographic units, typically a few hundred units, than in a nationwide surveillance context, in which a few thousands units may be involved.

The present study evaluates the performance of six cluster detection methods in the particular context of nationwide surveillance of a rare disease. Several single hot-spot cluster scenarios selected to approximate realistic situations were considered. The study focused on the elliptic and flexible scan methods, the double connected and maximum linkage spatial scan methods and the genetic algorithm based method, in addition to the circular scan method. The ability of each method to detect the presence of the true cluster and its ability to locate the cluster as precisely as possible were evaluated. Based on data from the French National Registry of Childhood Hematological Malignancies, the study provides new insights into the systematic investigation for clusters in the context of a rare disease distributed over a large territory.

## Methods

### Material

#### Geographic data

France consists of 22 *régions*, 96 *départements *and around 36500 *communes*, the smallest administrative units. In 2003, a new non-administrative division, the *living zone*, was created by the National Institute of Statistics and Economic Studies (INSEE) to describe the rural space in France. A *living zone *(LZ), which is comprised of several neighboring *communes*, is defined as the smallest territory in which people have access to employment and everyday facilities (e.g. supermarket, school, police station, post office, doctor, pharmacy, etc.). There are 1916 LZ, of which 1745 are located in the rural space. According to the last national census (table 1), the population of a LZ varied from 270 people to 9.8 million people (25th percentile = 6220, median = 9755, 75th percentile = 17968). Because 21 LZ are located on islands and thus disconnected from the main territory, they were not included. Thus, 1895 LZ were included in this national study.

**Table 1:** Number of *Communes*, area and population of the 1895 living zones (LZ) in France.

	Mean	Minimum	Q1	Median	Q3	Maximum
Number of *Communes *per LZ	19.1	1	7	13	24	556

Area (km^2^)	282.3	0.4	108.8	193	340.2	3863.2

Total population (1999)	30542.0	270	6219.8	9754.5	17 968	9802327

Population 0-14 years (1999)	5453.7	71	1068	1725	3298	1823195

AL incidence rate (per 100000 person-years)	40.8	0.0	0.0	36.5	58.6	545.9

Expected cases of AL per LZ (1990-2006)	4.1	0.1	0.8	1.3	2.4	1388.3

AL: childhood acute leukemia. Q1: first quartile. Q3: third quartile

#### Childhood acute leukemia data

This study, conducted in the context of a rare disease, was based on data from the French National Registry of Childhood Hematopoietic malignancies (NRCH) [8]. All cases of acute leukemia (AL) registered in the NRCH and diagnosed between 1990 and 2006 were included.

Each case was associated with the case's living zone of residence at the time of diagnosis. There were 7675 cases located in the 1895 LZ considered.

#### Population data

The age-specific populations of each *commune *were estimated from the 1999 census data and the annual population estimates on the *département *scale for the period 1990-2006 provided by the National Institute of Statistics and Economic Studies (INSEE). The annual populations of the *communes *were derived from the annual population estimates for the *départements *to which they belong, under the assumption that the proportions of the *commune *populations in the *départements *remained stable and equal to the corresponding proportions for census year 1999. The population of each LZ for the years 1990 to 2006 was then estimated as the sum of the populations of its *communes*.

The age-specific numbers of cases of childhood AL expected in each LZ under the hypothesis that the incidence rate was homogeneous were based on the population estimates and the national incidence rates provided by the NRCH for the whole period, 1990-2006. On average, 4.1 cases were expected in a LZ with a range of 0.1-1388.3 cases (table 1).

### Cluster detection methods

The performance of six cluster detection methods, all based on the likelihood ratio statistic developed by Kulldorff [1], were compared. Under the null hypothesis, the risks of AL within (p) and outside (q) a cluster zone *z *consisting of several connected LZ are equal, while p > q under the alternative hypothesis that z is a cluster zone. Thus, the likelihood ratio associated with z is LR_z _= ${\left(\frac{o_{z}}{e_{z}}\right)}^{o_{z}}{\left(\frac{O-o_{z}}{E-e_{z}}\right)}^{O-o_{z}}1\left\{\frac{o_{z}}{e_{z}}>\frac{O-o_{z}}{E-e_{z}}\right\}$, in which O and E are the numbers of observed and expected cases over the whole territory, and o_z _and e_z _the numbers of observed and expected cases in the cluster zone.

The test statistic LR (or its logarithm, LLR) is then defined as the maximum of the likelihood ratio function over the whole set of cluster candidates Z, and the connected area in which this maximum is achieved is defined as the most likely cluster. $\text{LR}=\underset{\text{z}\in \text{Z}}{\max}{\text{LR}}_{\text{z}}$

For each method, a maximum cluster size of 20 LZ was considered in the main analysis. The performances of the cluster detection methods with a 25 LZ limit and a 10 LZ limit were also investigated in an additional analysis.

The six methods under study mainly differ in terms of the manner in which the set Z is constructed.

#### Circular and elliptic scan methods [1,2]

The circular and elliptic scan methods implemented in the SaTScan software [9] were used. The circular scan method (*scan-c*) is based on a circular window that scans the whole territory moving from one LZ to the next.

In the elliptic scan method (*scan-e)*, the window is defined by the length of its semimajor axis, its shape (ratio between the semimajor and semiminor axes) and the angle between the horizontal line and its semimajor axis. For each semimajor axis length, the latter two parameters vary in order to cover a large territory. The likelihood ratio statistic LR can be penalized in order to favor compact clusters. The analyses were performed with no penalty and strong penalty, but only the results with no penalty (*scan-e0*) are presented herein.

##### - Flexible scan method [3]

**Figure 1:**
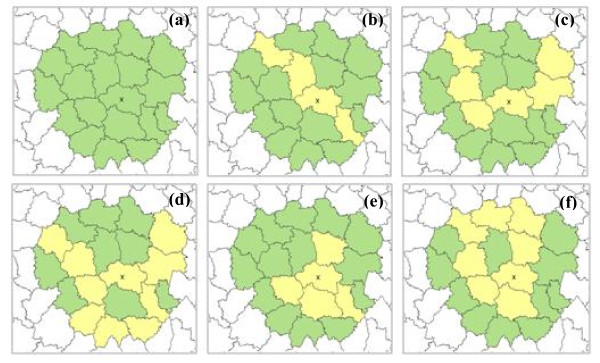
**Illustration of five cluster candidate areas in the flexible scan method **[3] . (a) Neighborhood of the cross unit. (b)-(f) 5 particular cluster candidate areas (in yellow) included in the neighborhood of the cross unit.

The flexible scan method, implemented in the FleXScan software [10], is based on an unvarying circular moving window, the size of which was set to 20 LZ, and considers not only the whole window as a cluster candidate but also all the connected areas included in it (Figure 1). Tango proposed restricting the log likelihood ratio LLR_z _in order to retain only areas made up of high-risk units as cluster candidates [11].

The main analyses in this study were performed with no restriction (noted *FleX*).

##### - Genetic algorithm method [7]

**Figure 2:**
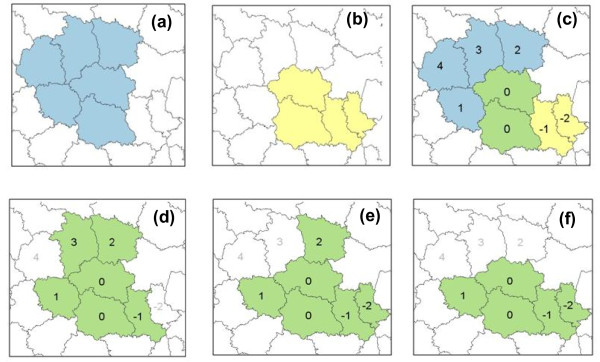
**Illustration of the genetic algorithm method - offspring resulting from the cross between two parent areas**. (a)-(b) the two parent areas. (c) cross between the two parent areas with units in parent 'a' and parent 'b' coded positively and negatively, respectively, and the intersection coded 0 (in green). (d)-(f) three offspring created using the genetic algorithm procedure (see [7]).

The genetic algorithm method was implemented using a C++ code provided by the authors. The genetic algorithm constructs successive sets of connected areas, called *generations *from the 1895 LZ on the basis of an improvement in the likelihood ratio from one step to the next. The first generation is created by adjoining a neighbor to each LZ while increasing the likelihood ratio without reaching the maximum size set at 20 LZ. Offspring areas are then created from repeated crosses between areas from that generation (Figure 2). The new generation is then made up of the areas of highest LLRz in the previous generation and its offspring. The last generation constitutes the panel Z of cluster candidates on which the test statistic is evaluated. Overall, 20 generations were created. The maximum number of "cross attempts" and "successful crosses" used by the algorithm to create new generations was set to 473 (one fourth of the LZ).

A strong non-compactness penalty was also used (referred to as *GA-1*).

##### - Dynamic minimum spanning tree methods (Costa MA et al., submitted)

The dynamic minimum spanning tree methods were applied using a program provided by the authors. The double connected spatial scan method (*Double*) and the maximum linkage scan method (*Mlink*) aim to create a set of cluster candidates by aggregating pre-selected neighbors with each LZ on the basis of different expansion criteria. The *Double *method imposes a double connection constraint so that the LZ that is adjoined at each step is the one that increases the LR function the most among all the neighboring LZ that are connected to at least two LZ in the current cluster. The algorithm stops when either the cluster candidate comprises 20 LZ or there is no candidate neighbor that makes the likelihood ratio increase. In the *Mlink *method the LZ that is adjoined is the one that maximizes the LR function among the LZ that are the most connected to the current cluster, i.e. among the neighboring LZ that have the highest number of connections compared to other neighbors. If no LZ is capable to increase the LR function, the Mlink method adjoins the LZ that decreases the least the LR function. The algorithm stops when the maximum cluster size of 20 LZ is attained.

### Alternative scenarios

**Figure 3:**
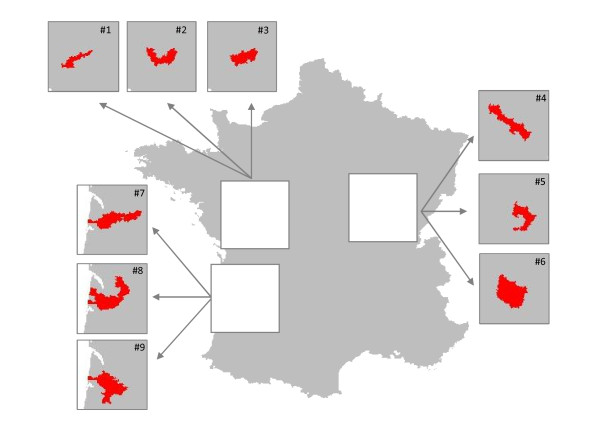
**The 9 clusters under study (3 cluster shapes and 3 cluster locations)**. The nine scenarios considered consisted in a combination of 3 cluster shapes (linear, U-shaped and compact) and 3 locations equivalent to 3 population sizes (20 expected cases for clusters 1-3,45 expected cases for clusters 4-6 and 115 expected cases for clusters 7-9).

Twenty-seven scenarios of alternative assumption (H1) that a single hot-spot cluster existed were considered. The scenarios consisted in a combination of 3 cluster shapes (linear, U-shape and compact), 3 locations equivalent to 3 population sizes (table 2 and Figure 3) and 3 relative risk values (RR = 1.5, 2.0, and 3.0). The linear clusters, which may be observed along rivers or roads, and the compact clusters which may arise around point-sources, were often considered in published studies ([2-4,7,11-17] and Costa MA, Assunção RM, Kulldorff M: Constrained spanning tree algorithms for irregularly shaped spatial clustering, submitted). The additional U-shape clusters were chosen because they seemed compatible, for instance, with territories around a lake or the mouth of a river. The expected numbers of cases of AL were about 20, 45 and 115 cases in the "small", "moderate" and "large" clusters, respectively, over a 17-year period.

**Table 2:** Description of the simulated alternative cluster scenarios

	**"Small Clusters"**^**1**^			**"Moderate Clusters"**^**1**^			**"Large Clusters"**^**1**^		
	
	#1	#2	#3	#4	#5	#6	#7	#8	#9
	Linear	U-shaped	Compact	Linear	U-shaped	Compact	Linear	U-shaped	Compact
	No. LZ = 6	No. LZ = 10	No. LZ = 8	No. LZ = 7	No. LZ = 7	No. LZ = 11	No. LZ = 12	No. LZ = 16	No. LZ = 13
***Population (1999, all ages)***									
Total	**147794**	**153067**	**136704**	**360752**	**351089**	**404176**	**948557**	**954460**	**913030**
Mean	24632.3	15306.7	17088.0	51536.0	50155.6	36743.3	79046.4	59653.8	76085.8
SD	35843.1	15350.6	16754.8	100719.4	101328.7	81488.4	223805.2	194750.3	224749.6
Min	6519	3963	6519	8267	7177	2949	5202	2259	4051
Max	97315	57355	57355	279841	279841	279841	788887	788887	788887
									
**Expected No. AL**	**20.8**	**21.4**	**19.7**	**44.9**	**44.0**	**50.1**	**115.4**	**116.8**	**113.6**

No. AL: number of childhood acute leukemia; No. LZ: number of living zones in the cluster

^1 ^"small", "moderate" and "large" clusters are clusters with about 20, 45 and 115 cases of childhood acute leukemia over the period 1990-2006, respectively.

Under the H1 hypothesis, the number of cases O_i _in LZ *i *follows a Poisson distribution with parameter ρ_i*_E_i_, the product of the expected number of cases Ei and ρ_i _the relative risk, equal to RR if *i *belongs to the cluster and one otherwise. For each of the 27 alternative scenarios, the cases were randomly allocated to each LZ conditionally on the total number of cases O observed over the whole territory from 1990 to 2006, from a multinomial distribution with parameters proportional to the expected numbers of cases.

### Statistical significance and estimation of power

For each method under study except one, 10000 Monte Carlo replications of the dataset were made under the null hypothesis (ρi = 1 for all LZ i), on the basis of a multinomial distribution of cases with parameters proportional to the expected numbers (reference dataset of the test statistic). For the *FleX *method with a 25-LZ window, 5000 Monte Carlo simulations were made. Then, 250 Monte Carlo replications were carried out for each of the 27 alternative scenarios in order to estimate the power of each method with a standard error of less than 5%. For each replicated dataset, the p-value was defined as the proportion of values from the reference dataset that were greater than or equal to the observed statistic.

In the present context of a systematic nationwide investigation for clusters, the decision was taken to promote sensitivity and thus to limit the probability of missing a true cluster, rather than avoiding false positive results. Therefore, all the tests were implemented with a significance level of 0.10.

### Power evaluation metrics

#### Usual power

The usual power was estimated as the proportion of the 250 alternative simulations that achieved statistical significance, irrespective of where the detected cluster was located.

#### Power to detect at least one LZ of the true cluster

The power to detect at least one LZ was defined as the proportion of simulations with a significant result and at least one true positive LZ, i.e., included in both the true and the detected clusters.

#### Power to detect exactly the "true" cluster ("exact" power)

The "exact" power was estimated as the proportion of simulations that enabled significant detection and exact location of the "true" cluster, i.e. without any false positive or missing LZ.

#### Average sensitivity

The average sensitivity was defined as the average proportion of LZ in the true cluster that was correctly detected, over the 250 simulations.

#### Average Positive Predictive Value (PPV)

The average positive predictive value was estimated as the average proportion of LZ in the detected cluster that belonged to the true cluster, over the 250 simulations.

#### Average cost

In line with Tango (2005), the average cost under the alternative situation in which a cluster of size s* existed was defined as C = C_1_*E(s*-S) + C_2_*E(L-s*), in which C_1 _is the cost of erroneously not including a LZ and C2 is the cost of erroneously including a LZ in the cluster, respectively, L and S are two random variables that represent the size of the detected cluster and the number of LZ correctly detected, and E() is the operator for an expected value.

C1 and C2 were both set to 1, so that the cost was the sum of the numbers of LZ missed and LZ erroneously included in the detected cluster.

### Computational time

All the methods were implemented on a Windows Dell R710 server (2.93 Ghz, RAM 32 Go), except the genetic algorithm, for which a Unix Dell R710 server (2.93 Ghz, RAM 64 Go) was used.

With a maximum cluster size window of 20 LZ, the typical running time for the 250 replications of a given cluster scenario, e.g. the small linear cluster with a relative risk of 1.5, ranged from 1 minute, for *Double*, to 3 hours, for *FleX *(additional file 1).

Although the running time remained quite stable for most of the methods when the maximum cluster size was increased to 25 LZ, it doubled for the *GA-1 *method and increased exponentially for the *FleX *method (3.2 days).

## Results

Table 3 shows an example of the evaluation metrics obtained on the basis of one of the 250 datasets generated for the 6th cluster. The latter was a compact cluster consisting of 11 LZ with 50 expected cases, and had a relative risk of 2. In this example, the methods evidenced a cluster sized between 8 LZ (with *Double*) and 18 LZ (with *Scan-c *and *Scan-e0*). The circular and elliptic scan methods detected large clusters including almost all of the true cluster, but also a large proportion of false positive LZ (PPV = 0.56 and 0.61 for *Scan-c *and *Scan-e0*, respectively). In contrast, the *Double *method detected a smaller cluster and had fewer false positive LZ (PPV = 0.75) but missed about half of the true cluster (sensitivity = 0.55). The *FleX *and *GA-1 *methods yielded intermediate results correctly detecting 8 and 9 LZ, respectively, with a PPV greater than 0.6. The *GA-1 *method minimized the cost, with 6 LZ either missed or erroneously detected.

**Table 3:** Performance of cluster detection methods on one replicated dataset.

	Scan-c	Scan-e0	FleX	GA-1	Double	Mlink
p-value	< 0.0001	< 0.0001	< 0.0001	< 0.0001	< 0.0001	< 0.0001
No. LZ	18	18	13	13	8	13
True Positive LZ^1^	10	11	8	9	6	7
Sensitivity^2^	0.91	1.00	0.73	0.82	0.55	0.64
PPV^3^	0.56	0.61	0.62	0.69	0.75	0.54
Cost^4^	9	7	8	6	7	10

Results for the sixth cluster scenario (compact cluster, E = 50.1 cases, covering 11 LZ), with a relative risk of 2.

Results based on 250 Monte Carlo replications. *Scan-c*: circular scan method, *Scan-e*0: elliptic scan method with no penalty, *FleX*: unrestricted flexible scan method, *GA-1*: strongly penalized genetic algorithm, *Double *and *Mlink*: dynamic minimum spanning tree method with double and maximum link connections, respectively. No. LZ: number of living zones in the detected cluster. ^1 ^number of living zones in the intersection of the true and detected clusters. ^2 ^sensitivity: proportion of living zones in the true cluster that are correctly detected. ^3 ^proportion of living zones in the detected cluster that are in the "true" cluster. ^4 ^number of living zones that are either missed or erroneously detected.

### Power

The estimated *usual power *of all the methods increased with the number of expected cases in the cluster and the relative risk (table 4). The *usual power *was greater than 0.8, and even greater than 0.9, for all the scenarios under study with a relative risk of 3, and, for the large and moderate clusters with a relative risk of at least 2. Only Mlink had *a usual power *of at least 0.8 to detect a small cluster with a relative risk of 2. Mlink performed better than the other methods with regard to the detection of small and moderate clusters with a relative risk of 1.5. Mlink also had the highest power (≥ 0.95) to detect large clusters with a relative risk of 1.5, which were detected by the other methods with a *usual power *of about 0.8 (slightly lower for *FleX*).

**Table 4:** *Usual power *of the cluster detection methods

		**"Small Clusters"**^**1**^			**"Moderate Clusters"**^**1**^			**"Large Clusters"**^**1**^		
	
		#1	#2	#3	#4	#5	#6	#7	#8	#9
		Linear	U-Shaped	Compact	Linear	U-Shaped	Compact	Linear	U-Shaped	Compact
		No LZ = 6	No. LZ = 10	No. LZ = 8	No. LZ = 7	No. LZ = 7	No. LZ = 11	No. LZ = 12	No. LZ = 16	No. LZ = 13
**RR = 1.5**	Scan-c	0.18	0.16	0.14	0.30	0.32	0.40	**0.84**	**0.85**	**0.83**
	Scan-e0	0.11	0.11	0.11	0.26	0.26	0.36	**0.83**	**0.83**	0.79
	FleX	0.12	0.15	0.16	0.25	0.29	0.36	0.78	0.74	0.76
	GA-1	0.14	0.11	0.10	0.33	0.30	0.44	**0.87**	**0.87**	**0.83**
	Double	0.14	0.13	0.13	0.26	0.25	0.39	**0.82**	0.79	0.78
	Mlink	0.65	0.60	0.60	0.71	0.73	0.79	**0.95**	**0.96**	**0.96**

**RR = 2.0**	Scan-c	0.46	0.49	0.52	**0.94**	**0.96**	**1.00**	**1.00**	**1.00**	**1.00**
	Scan-e0	0.51	0.51	0.50	**0.95**	**0.95**	**0.98**	**1.00**	**1.00**	**1.00**
	FleX	0.52	0.57	0.52	**0.93**	**0.92**	**0.97**	**1.00**	**1.00**	**1.00**
	GA-1	0.32	0.41	0.43	**0.95**	**0.95**	**1.00**	**1.00**	**1.00**	**1.00**
	Double	0.44	0.42	0.48	**0.92**	**0.90**	**0.98**	**1.00**	**1.00**	**1.00**
	Mlink	**0.83**	**0.82**	**0.85**	**1.00**	**1.00**	**1.00**	**1.00**	**1.00**	**1.00**

**RR = 3.0**	Scan-c	**0.97**	**0.99**	**0.99**	**1.00**	**1.00**	**1.00**	**1.00**	**1.00**	**1.00**
	Scan-e0	**0.99**	**0.99**	**0.99**	**1.00**	**1.00**	**1.00**	**1.00**	**1.00**	**1.00**
	FleX	**0.98**	**1.00**	**0.99**	**1.00**	**1.00**	**1.00**	**1.00**	**1.00**	**1.00**
	GA-1	**0.92**	**0.98**	**0.98**	**1.00**	**1.00**	**1.00**	**1.00**	**1.00**	**1.00**
	Double	**0.98**	**0.97**	**0.99**	**1.00**	**1.00**	**1.00**	**1.00**	**1.00**	**1.00**
	Mlink	**1.00**	**1.00**	**1.00**	**1.00**	**1.00**	**1.00**	**1.00**	**1.00**	**1.00**

*Scan-c*: circular scan method, *Scan-e*0: standard elliptic scan method, *FleX*: unrestricted flexible scan method, *GA-1*: strongly penalized genetic algorithm, *Double *and *Mlink*: dynamic minimum spanning tree method with double and maximum link connections, respectively. No. LZ: size of living zones in the cluster (number of living zones); RR: relative risk in the true cluster. Results based on 250 Monte Carlo replications.

^1 ^"small", "moderate" and "large" clusters are clusters with about 20, 45 and 115 cases of childhood acute leukemia over the period 1990-2006, respectively.

The *power to detect at least one LZ *in the true cluster was very similar to the *usual power*, but the highest power to detect a small cluster with a relative risk less than or equal to 2.0 (again obtained with Mlink) was less than 0.6 (table 5). The *power to detect at least one LZ *in the true cluster was particularly low for the small and moderate clusters with a relative risk of 1.5.

**Table 5:** *Power to detect at least one LZ *in the true cluster.

		**"Small Clusters"**^**1**^			**"Moderate Clusters"**^**1**^			**"Large Clusters"**^**1**^		
	
		#1	#2	#3	#4	#5	#6	#7	#8	#9
		Linear	U-Shaped	Compact	Linear	U-Shaped	Compact	Linear	U-Shaped	Compact
		No. LZ = 6	No. LZ = 10	No. LZ = 8	No. LZ = 7	No. LZ = 7	No. LZ = 11	No. LZ = 12	No. LZ = 16	No. LZ = 13
**RR = 1.5**	Scan-c	0.04	0.07	0.05	0.22	0.21	0.34	**0.83**	**0.82**	**0.80**
	Scan-e0	0.03	0.02	0.05	0.19	0.19	0.31	**0.81**	**0.81**	0.76
	FleX	0.04	0.07	0.07	0.17	0.19	0.28	0.76	0.72	0.72
	GA-1	0.02	0.05	0.02	0.24	0.23	0.36	**0.85**	**0.86**	**0.80**
	Double	0.03	0.06	0.05	0.17	0.16	0.32	**0.80**	0.76	0.74
	Mlink	0.11	0.13	0.11	0.34	0.34	0.50	**0.90**	**0.91**	**0.88**

**RR = 2.0**	Scan-c	0.38	0.41	0.44	**0.94**	**0.92**	**1.00**	**1.00**	**1.00**	**1.00**
	Scan-e0	0.43	0.39	0.46	**0.94**	**0.94**	**0.98**	**1.00**	**1.00**	**1.00**
	FleX	0.44	0.54	0.47	**0.92**	**0.91**	**0.97**	**1.00**	**1.00**	**1.00**
	GA-1	0.25	0.37	0.34	**0.94**	**0.92**	**0.99**	**1.00**	**1.00**	**1.00**
	Double	0.38	0.36	0.42	**0.91**	**0.88**	**0.98**	**1.00**	**1.00**	**1.00**
	Mlink	0.59	0.57	0.58	**0.96**	**0.96**	**0.99**	**1.00**	**1.00**	**1.00**

**RR = 3.0**	Scan-c	**0.97**	**0.99**	**0.99**	**1.00**	**1.00**	**1.00**	**1.00**	**1.00**	**1.00**
	Scan-e0	**0.99**	**0.95**	**0.99**	**1.00**	**1.00**	**1.00**	**1.00**	**1.00**	**1.00**
	FleX	**0.98**	**0.99**	**0.99**	**1.00**	**1.00**	**1.00**	**1.00**	**1.00**	**1.00**
	GA-1	**0.92**	**0.98**	**0.98**	**1.00**	**1.00**	**1.00**	**1.00**	**1.00**	**1.00**
	Double	**0.98**	**0.96**	**0.98**	**1.00**	**1.00**	**1.00**	**1.00**	**1.00**	**1.00**
	Mlink	**0.99**	**0.99**	**1.00**	**1.00**	**1.00**	**1.00**	**1.00**	**1.00**	**1.00**

*Scan-c*: circular scan method, *Scan-e*0: standard elliptic scan method, *FleX*: unrestricted flexible scan method, *GA-1*: strongly penalized genetic algorithm, *Double *and *Mlink*: dynamic minimum spanning tree method with double and maximum link connections, respectively. No. LZ: size of the cluster (number of living zones); RR: relative risk in the true cluster. Results based on 250 Monte Carlo replications.

^1 ^"small", "moderate" and "large" clusters are clusters with about 20, 45 and 115 cases of childhood acute leukemia over the period 1990-2006, respectively.

Lastly, none of the methods was ever able to detect the underlying cluster *exactly*, irrespective of the population size, shape or relative risk of the cluster.

### Sensitivity, positive predictive value and cost

Table 6 reports the results for the scenarios in which at least one LZ in the true cluster was detected with a power of about 0.8 by most of the methods.

**Table 6:** Average sensitivity, PPV and cost of cluster detection methods

		**"Small Clusters"**^**1**^			**"Moderate Clusters"**^**1**^			**"Large Clusters"**^**1**^		
	
		#1	#2	#3	#4	#5	#6	#7	#8	#9
		Linear	U-Shaped	Compact	Linear	U-Shaped	Compact	Linear	U-Shaped	Compact
		6 LZ	10 LZ	8 LZ	7 LZ	7 LZ	11 LZ	12 LZ	16 LZ	13 LZ
**Average sensitivity**										
RR = 1.5	Scan-c							0.52	0.40	0.68
	Scan-e0							0.52	0.38	0.61
	FleX							0.43	0.35	0.49
	GA-1							0.37	0.30	0.47
	Double							0.42	0.31	0.39
	Mlink							0.49	0.38	0.46

RR = 2.0	Scan-c				0.49	0.63	0.81	0.59	0.49	0.82
	Scan-e0				0.64	0.46	0.75	0.71	0.45	0.78
	FleX				0.57	0.68	0.64	0.50	0.43	0.64
	GA-1				0.54	0.54	0.78	0.45	0.35	0.60
	Double				0.48	0.48	0.63	0.50	0.37	0.51
	Mlink				0.50	0.54	0.69	0.58	0.46	0.57

RR = 3.0	Scan-c	0.48	0.43	0.79	0.51	0.75	0.93	0.63	0.54	0.90
	Scan-e0	0.82	0.18	0.82	0.85	0.50	0.92	0.89	0.49	0.88
	FleX	0.68	0.70	0.82	0.69	0.86	0.78	0.61	0.51	0.75
	GA-1	0.65	0.60	0.73	0.53	0.57	0.86	0.57	0.39	0.70
	Double	0.43	0.39	0.81	0.52	0.57	0.79	0.60	0.44	0.59
	Mlink	0.44	0.44	0.66	0.55	0.63	0.81	0.66	0.49	0.62

**Average PPV**										
RR = 1.5	Scan-c							0.46	0.50	0.69
	Scan-e0							0.40	0.39	0.53
	FleX							0.45	0.49	0.57
	GA-1							0.47	0.52	0.58
	Double							0.54	0.54	0.62
	Mlink							0.43	0.46	0.51

RR = 2.0	Scan-c				0.39	0.46	0.81	0.57	0.57	0.84
	Scan-e0				0.47	0.33	0.65	0.61	0.52	0.73
	FleX				0.45	0.49	0.67	0.57	0.61	0.74
	GA-1				0.42	0.41	0.77	0.68	0.69	0.76
	Double				0.56	0.55	0.77	0.70	0.67	0.80
	Mlink				0.50	0.51	0.75	0.63	0.59	0.74

RR = 3.0	Scan-c	0.51	0.68	0.73	0.43	0.59	0.90	0.68	0.63	0.92
	Scan-e0	0.73	0.25	0.73	0.73	0.44	0.90	0.73	0.53	0.89
	FleX	0.57	0.76	0.77	0.65	0.70	0.83	0.75	0.79	0.88
	GA-1	0.55	0.79	0.82	0.52	0.55	0.89	0.88	0.86	0.86
	Double	0.72	0.73	0.88	0.70	0.73	0.92	0.85	0.81	0.89
	Mlink	0.65	0.66	0.80	0.68	0.72	0.91	0.82	0.70	0.91

**Average cost**										
RR = 1.5	Scan-c							14.00	16.70	8.60
	Scan-e0							15.50	19.80	12.50
	FleX							13.20	16.20	11.30
	GA-1							13.30	16.10	11.40
	Double							11.60	15.60	11.40
	Mlink							14.40	17.40	13.60

RR = 2.0	Scan-c				10.30	8.40	4.80	11.10	14.60	4.80
	Scan-e0				9.20	11.70	8.10	9.30	16.00	6.90
	FleX				8.30	7.40	7.60	10.70	13.60	7.70
	GA-1				9.20	9.50	5.30	9.60	13.40	7.70
	Double				7.00	6.90	6.60	9.10	13.40	8.40
	Mlink				8.50	8.00	6.60	10.10	14.80	9.10

RR = 3.0	Scan-c	8.00	9.10	4.50	9.10	5.70	2.10	8.70	12.70	2.40
	Scan-e0	3.60	13.80	4.60	3.70	8.50	2.30	5.50	15.40	3.10
	FleX	5.50	5.50	3.80	5.20	3.80	4.30	7.20	10.10	4.70
	GA-1	6.00	6.10	3.70	7.20	6.60	2.90	6.20	11.00	5.50
	Double	5.10	8.10	2.80	5.20	4.60	3.30	6.40	11.00	6.40
	Mlink	5.90	9.10	4.80	5.30	4.60	3.20	6.40	12.60	6.10

*Scan-c*: circular scan method, *Scan-e*0: standard elliptic scan method, *FleX*: unrestricted flexible scan method, *GA-1*: strongly penalized genetic algorithm, *Double *and *Mlink*: dynamic minimum spanning tree method with double and maximum link connections, respectively. Results based on 250 Monte Carlo replications. ^1 ^"small", "moderate" and "large" clusters are clusters with about 20, 45 and 115 cases of childhood acute leukemia over the period 1990-2006, respectively.

In all the corresponding categories of cluster population size and relative risk, the compact clusters were detected with a higher *average sensitivity *than the other clusters and the *Scan-c *and *Scan-e0 *methods performed best. *Scan-e*0 was the most sensitive for detection of linear clusters. In the small and moderate clusters, *FleX *detected the linear clusters better than all the other methods except *Scan-e*0, but was clearly the most sensitive for the U-shaped clusters. Few differences in the average *sensitivity *were observed for the detection of large U-shaped clusters.

Similarly, the *average positive predictive *value (PPV) was higher for the compact clusters, and better with the *Scan-c *method, except in the small cluster with RR = 3. The average PPV associated with the detection of linear and U-shaped clusters was of the same order of magnitude and none of the methods performed significantly better than the others. The *Double *method had, however, an average PPV greater than 0.7 in all the linear and U-shaped cluster scenarios, except the moderate cluster with RR = 2.0, for which its average PPV was higher than for other methods, but only about 0.55. The *Scan-e*0 method also had a good average PPV (0.73) for linear clusters with RR = 3.0 but appeared systematically with the lowest average values for U-shaped clusters, irrespective of the relative risk. Compact clusters were detected with an average PPV of about 0.7-0.8 when RR = 2.0. The maximum average PPV were obtained with the *Scan-c *method. With a relative risk of 3.0, the methods were quite similar and most of the average PPV were greater than 0.8.

The detection of the large clusters with RR = 1.5 was associated with an average cost of more than 10 LZ and even more than 15 LZ for the U-shaped cluster, except for the compact cluster detection with *Scan-c*, which resulted in an average cost of 8.6 LZ. More generally, the detection of large or moderate clusters was less costly for compact clusters and in those cases *Scan-c *was mostly associated with the lowest costs. The average costs for U-shaped clusters were higher than for other shapes. The highest values were systematically observed with the *Scan-e*0 method. The latter method was, however, more cost-effective for the detection of linear clusters with RR = 3.0.

### Additional analyses

#### Non-compactness penalty and restriction (additional file 2)

Irrespective of the cluster configuration, the elliptic scan method gave quite similar results for all evaluation metrics, with and without a penalty.

The flexible scan method with a restriction appeared to be as powerful as the standard flexible scan method with regard to the detection of a cluster and identification of at least one of its LZ. However, clusters were less precisely located with the restriction; the average sensitivity was systematically lower than with the unrestricted method.

In all the cluster scenarios, the genetic algorithm with no penalty tended to detect clusters as large as the maximum cluster size allowed, i.e. 20 LZ, while the detected clusters were about half the size with the non-compactness penalty. The average sensitivity was lower in most cases and the average positive predictive value systematically higher with the penalty. Irrespective of the cluster scenario, the average cost was also far greater when no penalty was considered. Incidentally, despite smaller detected clusters, the genetic algorithm with a strong penalty had a higher average sensitivity for the detection of moderate compact clusters, irrespective of the relative risk.

#### Maximum cluster sizes of 25 LZ and 10 LZ

When the maximum cluster size was increased to 25 LZ the results were similar to those obtained with a limit of 20 LZ (not shown).

While the *usual power *and the *power to detect at least one LZ *of the true cluster remained unchanged with a window of at most 10 LZ, the average *sensitivity *of all the methods decreased and their PPV tended to increase, particularly for the detection of large clusters (additional files 3 and 4). For small and moderate clusters, the greatest differences were observed with the *FleX *method, which became as sensitive as the other methods with regard to the detection of U-shaped clusters, with, however, a higher PPV. *Scan-e0 *remained the most sensitive method to detect linear clusters. Overall, the shift from 20 LZ to 10 LZ did not change the results of the genetic algorithm method.

## Discussion

The present study evaluated the performance of six cluster detection methods, the most famous of which was the widely used circular scan method [1], in several realistic alternative scenarios of a single hot-spot cluster of a rare disease, childhood AL, in mainland France.

Three situations emerged from the power study. (1) The less detectable clusters, i.e. the small clusters with a relative risk of 1.5 or 2.0 and the moderate clusters with a relative risk of 1.5: in these scenarios, the usual statistical power was mostly lower than 0.5 and all the cluster detection methods most often failed to detect at least one unit of the true cluster. (2) The large clusters with a relative risk of 1.5: in this case, all the methods detected at least one living zone 8 times out of 10. However, when the true cluster was linear or U-shaped, at most half of it was detected and a great number of living zones were misclassified, particularly with the elliptic scan method. All the methods and particularly the circular scan method performed slightly better in compact cluster detection. (3) The moderate and large clusters with a relative risk of 2.0 and all the clusters with a relative risk of 3.0 were far easier to detect and in most cases at least one living zone of the true cluster was detected. In particular, compact clusters were well detected by all the methods. In terms of sensitivity, positive predictive value and average cost, the elliptic scan method with no penalty detected the linear clusters better, while the flexible scan method without restriction located the U-shaped clusters more precisely than did the other methods.

All the methods require prior specification of the maximum cluster size. The published cluster detection studies often considered half of the total population, which is not realistic in a nationwide study. In this study, the parameter was therefore defined in terms of geographic units rather than population proportion. The influence of the parameter on power could not be readily assessed since increasing the size increased the computational time and did so exponentially with the unrestricted flexible scan method. In consequence, the maximum size was limited to 25 LZ. The shift from 20 to 25 LZ did not enable enhanced detection of small clusters with a relative risk of 1.5 or 2.0 or change the performance of the methods with regard to the detection of clusters with a relative risk of 3. Most of the results obtained with maximum cluster sizes of 10 LZ and 20 LZ were qualitatively similar, although the average sensitivity decreased. However, the flexible scan method, which exhibited the greatest decreases in sensitivity, was no longer superior to the other methods with regard to the detection of U-shaped clusters.

The restricted flexible scan method, with a local significance threshold set to the default value of 0.20, was as powerful as the method with no restriction with regard to detection of at least one LZ of the true cluster, but the average sensitivity was lower. However, the default parameter value may not be appropriate in the context of this study and the possibility of the restricted approach performing better with another value cannot be ruled out.

Several tuning parameters also hinder the use of the genetic algorithm method. No analysis has yet been done to determine which values would be recommended for a dataset as large as that used in the present study. The parameters were thus arbitrarily set to values that enabled varied generations in a reasonable computational time. Under those conditions, the genetic algorithm method with no penalty tended to detect large "octopus shaped" clusters, while the strongly penalized approach detected smaller clusters, but had a lower sensitivity than the elliptic or flexible scan methods.

Kulldorff et al. [2] reported that the elliptic scan method with a strong penalty was as powerful as the non-penalized method with regard to usual power. The finding was similar to that reported herein. However, no information on detected cluster locations was provided. In contrast, Costa et al. concluded recently that the method with no penalty was more powerful for detecting small irregular clusters (Costa MA, Assunção RM, Kulldorff M: Constrained spanning tree algorithms for irregularly shaped spatial clustering, submitted). However, even though the approach yielded a numerically higher power for small irregular clusters, few changes were observed when a penalty was added so that we would have rather concluded that the penalized and non-penalized results were similar.

In the last 10 years, several power studies involving at least one of the present methods have been carried out and most of them enabled comparison of cluster or clustering methods to the circular scan method.

Five studies evaluated the performance of the methods considering only the usual power [2,7,14-16], which may lead to erroneous conclusions due to false positive results. In this study, by far the highest usual power to detect small clusters with a relative risk of 1.5 or 2.0 was that of *Mlink*, but most of the time the detected cluster did not intersect the true cluster. In line with some other studies ([3,4,11-13,18,19] and Costa MA, Assunção RM, Kulldorff M: Constrained spanning tree algorithms for irregularly shaped spatial clustering, submitted), this study focused on the ability of each method not only to detect the presence of the true cluster but also to capture as many of its LZ as possible.

The previously published studies had various designs (additional file 5). Except for one of the two nationwide studies, all the studies covered a territory of less than 500 geographic units. In the context of a systematic investigation for clusters over a large territory, the power to detect a true cluster is reduced due to the large number of cluster candidates considered by the cluster detection method. The significance level was set to 0.10 in the simulation study, instead of the usual value of 0.05, so as to limit the lack of power. The present study led to results of the same order of magnitude as those of other published studies for comparable scenarios. The observed differences versus published studies are more likely to be explained by the population size and the relative risk of the true cluster than by the choice of the alpha-level. Half of the studies used publicly available datasets that were simulated in a rare disease context and considered the presence of a single circular cluster centered either on a rural, urban or mixed rural/urban county in the northeastern United States ([2,7,13,15,16] and Costa MA, Assunção RM, Kulldorff M: Constrained spanning tree algorithms for irregularly shaped spatial clustering, submitted). Some irregularly shaped cluster alternative scenarios defined in [14] were sometimes considered additionally ([7] and Costa MA, Assunção RM, Kulldorff M: Constrained spanning tree algorithms for irregularly shaped spatial clustering, submitted). Three other studies were conducted on 113 regions in the area of the Tokyo metropolis and Kanagawa Prefecture in Japan [3,11,17]. The nationwide study conducted in the United States focused on multiple cluster scenarios with several cancer sites [18]. Several methods were considered but the powers of the circular and elliptic scan methods were not evaluated and, due to the considerable computational time, the flexible scan method was finally applied on a larger scale (49 States). The great heterogeneity of the literature renders between-published study comparisons and comparison of the published results with those reported herein difficult. In contrast to the present study, many authors defined the relative risks in cluster areas so that the probability of rejecting the null hypothesis with a standard binomial test would be 0.999 if the cluster location were known a priori ([2,4,7,13-16] and Costa MA, Assunção RM, Kulldorff M: Constrained spanning tree algorithms for irregularly shaped spatial clustering, submitted). A relative risk of 5 was sometimes considered additionally ([4] and Costa MA, Assunção RM, Kulldorff M: Constrained spanning tree algorithms for irregularly shaped spatial clustering, submitted). This choice resulted in scenarios, in which cluster detection methods yielded the best performance. In the present context, the approach would lead to relative risks equal to 2.4, 1.9 and 1.5 in the small, moderate and large clusters, respectively. Those values are close to the values of 1.5, 2.0 and 3.0 considered in the present study. The alternative scenarios defined with that approach would thus have led to similar results. On the other hand, a relative risk of 1.5 in an area with about 20 expected cases would yield a local power of 0.68 for a local binomial test, markedly below the 0.999 threshold. The existence of such a less detectable cluster has not been considered as an alternative scenario in other published studies. Although less likely to be evidenced by a local test than the clusters considered in Kulldorff *et al*. [15], such clusters constitute, however, realistic scenarios. The large number of geographic units considered in the present study and consequently the large number of cluster candidates may have limited the ability of the study to detect small clusters.

## Conclusions

The present study showed that none of the circular scan or other recent sophisticated methods was powerful enough to detect and locate some realistic hot-spot clusters (E ≤ 45 and RR = 1.5). In less demanding scenarios, the methods differed in their ability to locate the true cluster: the elliptic scan window performed better in linear and compact cluster detection while the flexible scan method was superior for U-shaped clusters.

The context of this study was childhood leukemia in France. However, the authors believe that the results hold for any situation in which a systematic search for a localized cluster of a rare disease is conducted over a large territory. In such contexts, the elliptic scan method and flexible scan method, both of which are easy to use thanks to the SaTScan [9] and FleXScan [10] public software, seem the most able to detect clusters.

## Competing interests

The authors declare that they have no competing interests.

## Authors' contributions

SGB, DH and JC conceptualized the study design. AGG provided the data on childhood leukemia. SGB and CD wrote the programs to get the results of the simulations. CD carried out the simulations and interpreted the results with SGB. SGB wrote the first draft of the manuscript. All the authors participated in the writing of the final version and approved it.

## Supplementary Material

Additional file 1 **Computational time for analyzing 250 replicated datasets of a given cluster scenario, by maximum cluster size (10, 20, 25 LZ)**. For each of the 27 cluster scenarios, 250 replicated datasets were analyzed. The table gives the average running time for the 250 replications, and for one replication, of a given scenario. Click here for file

Additional file 2 **Performance of the *Elliptic scan *method with and without a penalty, the *Flexible scan *method with and without a restriction and the *Genetic Algorithm *with and without a non-compactness penalty**. Evaluation of the performance of each method, with and without restriction or penalty, for the 9 cluster scenarios with a relative risk of 2.0. Click here for file

Additional file 3 ***Power to detect at least one LZ *of the true cluster with a maximum cluster size of 10 LZ**. Power of each method to detect at least one LZ of the true cluster for the 27 cluster scenarios, based on 250 Monte Carlo replications for each. Click here for file

Additional file 4 **Average sensitivity, PPV and cost with a maximum cluster size of 10 LZ**. Estimation of the average sensibility, PPV and cost for each of the 27 cluster scenarios, based on 250 Monte Carlo replications. Click here for file

Additional file 5 **Study design of published studies on the performance of cluster detection methods**. Information on the methods, the study area, the cluster scenarios and the evaluation metrics considered in the published studies. Click here for file
