# Supplementary material for: Detection of clusters of a rare disease over a large territory: performance of cluster detection methods
Source: Int J Health Geogr. 2011 Oct 4;10:53. doi: 10.1186/1476-072X-10-53 (PMC3204219; doi:10.1186/1476-072X-10-53)
Supplement: Additional file 1 — Computational time for analyzing 250 replicated datasets of a given cluster scenario, by maximum cluster size (10, 20, 25 LZ). For each of the 27 cluster scenarios, 250 replicated datasets were analyzed. The table gives the average running time for the 250 replications, and for one replication, of a given scenario. [file 1476-072X-10-53-S1.PDF]

Additional file 1 - Computational time[1] for analyzing 250 replicated datasets of a given cluster scenario, by maximum cluster size (10, 20, 25 LZ)

	Maximum cluster size	Scan-c	Scan-e0	FleX	GA-1	Double	Mlink
250 replicated datasets	10 LZ	10 min	2 h	11 min	50 min	1 min	15 min
	20 LZ	11 min	2 h	3 h 7 min	2 h 40 min	1 – 2 min	52 min
	25 LZ	11 min	2 h	3.2 days	3 h – 6 h 15 min	1 – 2 min	52 min
One replicated dataset	10 LZ	2.4 s	28.8 s	2.6 s	12.0 s	0.2 s	3.6 s
	20 LZ	2.6 s	28.8 s	44.9 s	38.4 s	0.2 s - 0.4 s	12.5 s
	25 LZ	2.6 s	28.8 s	1116 s	43.2 s – 90 s	0.2 s - 0.4 s	12.5 s

[1] A Dell R710 server was used.

LZ: living zone. *Scan-c*: circular scan method, *Scan-e*0: standard elliptic scan method, *FleX*: unrestricted flexible scan method, *GA-1*: strongly penalized genetic algorithm, *Double* and *Mlink*: dynamic minimum spanning tree method with double and maximum link connections, respectively.
