# Supplementary material for: Detection of clusters of a rare disease over a large territory: performance of cluster detection methods
Source: Int J Health Geogr. 2011 Oct 4;10:53. doi: 10.1186/1476-072X-10-53 (PMC3204219; doi:10.1186/1476-072X-10-53)
Supplement: Additional file 2 — Performance of the Elliptic scan method with and without a penalty, the Flexible scan method with and without a restriction and the Genetic Algorithm with and without a non-compactness penalty. Evaluation of the performance of each method, with and without restriction or penalty, for the 9 cluster scenarios with a relative risk of 2.0. [file 1476-072X-10-53-S2.PDF]

Additional file 2 - *Elliptic scan* method with and without a penalty, *Flexible scan* method with and without a restriction and *Genetic Algorithm* with and without a non-compactness penalty

	"Small Clusters"						"Moderate Clusters"						"Large Clusters"					
	#1		#2		#3		#4		#5		#6		#7		#8		#9	
	Linear		U-Shaped		Compact		Linear		U-Shaped		Compact		Linear		U-Shaped		Compact	
	No. LZ = 6		No. LZ = 10		No. LZ = 8		No. LZ = 7		No. LZ = 7		No. LZ = 11		No. LZ = 12		No. LZ = 16		No. LZ = 13	
**Scan-e**	Penalty						Penalty						Penalty					
	No	Yes	No	Yes	No	Yes	No	Yes	No	Yes	No	Yes	No	Yes	No	Yes	No	Yes
Detected cluster size	10.00	9.30	10.00	9.30	11.10	9.60	11.10	10.30	11.10	10.30	13.50	12.50	14.40	13.30	14.40	13.30	14.30	13.40
Usual power	0.51	0.54	0.51	0.54	0.50	0.57	0.95	0.95	0.95	0.95	0.98	0.99	1.00	1.00	1.00	1.00	1.00	1.00
"At least one LZ" power	0.43	0.44	0.39	0.38	0.46	0.50	0.94	0.94	0.94	0.94	0.98	0.99	1.00	1.00	1.00	1.00	1.00	1.00
Average sensitivity	0.49	0.44	0.20	0.23	0.63	0.62	0.64	0.56	0.46	0.43	0.75	0.81	0.71	0.60	0.45	0.43	0.78	0.84
Average PPV[1]	0.38	0.37	0.24	0.27	0.54	0.60	0.47	0.44	0.33	0.35	0.65	0.75	0.61	0.59	0.52	0.53	0.73	0.84
Average cost	10.10	10.10	16.10	14.70	9.10	7.70	9.20	9.40	11.70	11.30	8.10	5.70	9.30	10.90	16.00	15.50	6.90	4.60
**FleX**	Restriction						Restriction						Restriction					
	No	Yes	No	Yes	No	Yes	No	Yes	No	Yes	No	Yes	No	Yes	No	Yes	No	Yes
Detected cluster size	9.20	6.10	9.60	7.00	9.40	6.80	9.30	6.60	9.90	6.70	10.60	8.40	10.70	7.50	11.20	7.90	11.40	8.40
Usual power	0.52	0.47	0.57	0.49	0.52	0.48	0.93	0.94	0.92	0.91	0.97	0.97	1.00	1.00	1.00	1.00	1.00	1.00
"At least one LZ" power	0.44	0.40	0.54	0.45	0.47	0.42	0.92	0.92	0.91	0.89	0.97	0.97	1.00	1.00	1.00	1.00	1.00	1.00
Average sensitivity	0.44	0.35	0.51	0.40	0.60	0.50	0.57	0.53	0.68	0.51	0.64	0.54	0.50	0.42	0.43	0.34	0.64	0.51
Average PPV[1]	0.31	0.40	0.55	0.61	0.54	0.62	0.45	0.59	0.49	0.55	0.67	0.72	0.57	0.67	0.61	0.70	0.74	0.79
Average cost	9.90	7.90	9.40	8.90	7.80	6.80	8.30	6.30	7.40	6.60	7.60	7.50	10.70	9.50	13.60	13.00	7.70	8.10
**Genetic algorithm GA**	Penalty						Penalty						Penalty					
	No	Yes	No	Yes	No	Yes	No	Yes	No	Yes	No	Yes	No	Yes	No	Yes	No	Yes
Detected cluster size	19.60	9.50	19.70	9.50	19.70	9.20	19.60	9.80	19.70	10.00	19.70	11.50	19.50	8.50	19.80	8.70	19.70	10.30
Usual power	0.47	0.32	0.44	0.41	0.45	0.43	0.92	0.95	0.88	0.95	0.96	1.00	1.00	1.00	1.00	1.00	1.00	1.00
"At least one LZ" power	0.40	0.25	0.40	0.37	0.39	0.34	0.92	0.94	0.86	0.92	0.96	0.99	1.00	1.00	1.00	1.00	1.00	1.00
Average sensitivity	0.52	0.41	0.62	0.47	0.60	0.49	0.68	0.54	0.62	0.54	0.57	0.78	0.60	0.45	0.52	0.35	0.56	0.60
Average PPV[1]	0.16	0.29	0.32	0.55	0.25	0.52	0.25	0.42	0.22	0.41	0.32	0.77	0.37	0.68	0.42	0.69	0.38	0.76
Average cost	19.30	10.60	17.30	10.10	18.10	9.30	17.10	9.20	18.00	9.50	18.30	5.30	17.00	9.60	19.10	13.40	18.00	7.70

[1] PPV : positive predictive value
