# Supplementary material for: Detection of clusters of a rare disease over a large territory: performance of cluster detection methods
Source: Int J Health Geogr. 2011 Oct 4;10:53. doi: 10.1186/1476-072X-10-53 (PMC3204219; doi:10.1186/1476-072X-10-53)
Supplement: Additional file 3 — Power to detect at least one LZ of the true cluster with a maximum cluster size of 10 LZ. Power of each method to detect at least one LZ of the true cluster for the 27 cluster scenarios, based on 250 Monte Carlo replications for each. [file 1476-072X-10-53-S3.PDF]

Additional file 3 - *Power to detect at least one LZ* of the true cluster with a maximum cluster size of 10 LZ

		"Small Clusters"			"Moderate Clusters"			"Large Clusters"		
		#1	#2	#3	#4	#5	#6	#7	#8	#9
		Linear	U-Shaped	Compact	Linear	U-Shaped	Compact	Linear	U-Shaped	Compact
		No. LZ = 6	No. LZ = 10	No. LZ = 8	No. LZ = 7	No. LZ = 7	No. LZ = 11	No. LZ = 12	No. LZ = 16	No. LZ = 13
**RR = 1.5**	Scan-c	0.03	0.06	0.04	0.18	0.18	0.30	**0.82**	0.80	0.78
	Scan-e0	0.04	0.03	0.05	0.20	0.19	0.28	0.77	0.77	0.74
	FleX	0.04	0.06	0.05	0.17	0.18	0.26	0.79	0.77	0.75
	GA-1	0.03	0.04	0.03	0.23	0.21	0.34	**0.86**	**0.88**	**0.82**
	Double	0.03	0.07	0.04	0.18	0.17	0.27	0.78	0.77	0.76
	Mlink	0.10	0.11	0.09	0.29	0.29	0.46	**0.88**	**0.87**	**0.86**
**RR = 2.0**	Scan-c	0.38	0.38	0.41	**0.94**	**0.91**	**0.98**	**1.00**	**1.00**	**1.00**
	Scan-e0	0.46	0.41	0.46	**0.93**	**0.93**	**0.97**	**1.00**	**1.00**	**1.00**
	FleX	0.42	0.43	0.43	**0.94**	**0.91**	**0.96**	**1.00**	**1.00**	**1.00**
	GA-1	0.34	0.38	0.35	**0.95**	**0.91**	**0.99**	**1.00**	**1.00**	**1.00**
	Double	0.43	0.38	0.42	**0.93**	**0.90**	**0.94**	**1.00**	**1.00**	**1.00**
	Mlink	0.59	0.54	0.58	**0.96**	**0.95**	**0.99**	**1.00**	**1.00**	**1.00**
**RR = 3.0**	Scan-c	**0.96**	**0.98**	**0.99**	**1.00**	**1.00**	**1.00**	**1.00**	**1.00**	**1.00**
	Scan-e0	**0.98**	**0.95**	**0.99**	**1.00**	**1.00**	**1.00**	**1.00**	**1.00**	**1.00**
	FleX	**0.98**	**0.98**	**0.99**	**1.00**	**1.00**	**1.00**	**1.00**	**1.00**	**1.00**
	GA-1	**0.94**	**0.98**	**0.98**	**1.00**	**1.00**	**1.00**	**1.00**	**1.00**	**1.00**
	Double	**0.98**	**0.97**	**0.98**	**1.00**	**1.00**	**1.00**	**1.00**	**1.00**	**1.00**
	Mlink	**0.99**	**1.00**	**1.00**	**1.00**	**1.00**	**1.00**	**1.00**	**1.00**	**1.00**

Scan-c: circular scan method, *Scan-e*0: standard elliptic scan method, *FleX*: unrestricted flexible scan method, *GA-1*: strongly penalized genetic algorithm, *Double* and *Mlink*: dynamic minimum spanning tree method with double and maximum link connections, respectively. No. LZ: number of living zones in the cluster; RR: relative risk in the true cluster. Bold indicates figures greater than or equal to 0.8.
