# Supplementary material for: Detection of clusters of a rare disease over a large territory: performance of cluster detection methods
Source: Int J Health Geogr. 2011 Oct 4;10:53. doi: 10.1186/1476-072X-10-53 (PMC3204219; doi:10.1186/1476-072X-10-53)
Supplement: Additional file 4 — Average sensitivity, PPV and cost with a maximum cluster size of 10 LZ. Estimation of the average sensibility, PPV and cost for each of the 27 cluster scenarios, based on 250 Monte Carlo replications. [file 1476-072X-10-53-S4.PDF]

Additional file 4 - Average sensitivity, PPV and cost with a maximum cluster size of 10 LZ

		"Small Clusters"			"Moderate Clusters"			"Large Clusters"		
		#1 Linear 6 LZ	#2 U-Shaped 10 LZ	#3 Compact 8 LZ	#4 Linear 7 LZ	#5 U-Shaped 7 LZ	#6 Compact 11 LZ	#7 Linear 12 LZ	#8 U-Shaped 16 LZ	#9 Compact 13 LZ
**Average sensitivity**										
RR = 1.5	Scan-c							0.37	0.24	0.50
	Scan-e0							0.41	0.28	0.47
	FleX							0.31	0.21	0.38
	GA-1							0.35	0.27	0.42
	Double							0.30	0.20	0.33
	Mlink							0.37	0.25	0.36
RR = 2.0	Scan-c				0.44	0.56	0.73	0.49	0.27	0.65
	Scan-e0				0.60	0.42	0.64	0.52	0.32	0.62
	FleX				0.45	0.54	0.59	0.39	0.26	0.51
	GA-1				0.51	0.50	0.68	0.45	0.34	0.55
	Double				0.45	0.44	0.48	0.37	0.27	0.42
	Mlink				0.47	0.51	0.61	0.52	0.33	0.49
RR = 3.0	Scan-c	0.36	0.36	0.68	0.47	0.66	0.86	0.58	0.30	0.72
	Scan-e0	0.81	0.17	0.74	0.83	0.50	0.85	0.64	0.35	0.73
	FleX	0.42	0.38	0.62	0.52	0.69	0.73	0.49	0.29	0.57
	GA-1	0.58	0.61	0.75	0.54	0.58	0.79	0.56	0.38	0.64
	Double	0.42	0.37	0.76	0.52	0.55	0.51	0.52	0.36	0.49
	Mlink	0.43	0.37	0.65	0.53	0.62	0.75	0.63	0.37	0.59
**Average PPV**										
RR = 1.5	Scan-c							0.56	0.52	0.77
	Scan-e0							0.54	0.50	0.68
	FleX							0.56	0.56	0.74
	GA-1							0.53	0.56	0.63
	Double							0.59	0.57	0.67
	Mlink							0.56	0.53	0.62
RR = 2.0	Scan-c				0.43	0.50	0.92	0.73	0.59	0.95
	Scan-e0				0.57	0.39	0.78	0.71	0.59	0.86
	FleX				0.59	0.64	0.92	0.79	0.71	0.93
	GA-1				0.43	0.43	0.81	0.75	0.73	0.79
	Double				0.59	0.58	0.81	0.74	0.72	0.82
	Mlink				0.55	0.56	0.82	0.76	0.69	0.82
RR = 3.0	Scan-c	0.58	0.76	0.81	0.47	0.59	0.99	0.83	0.59	0.99
	Scan-e0	0.77	0.25	0.80	0.76	0.46	0.97	0.84	0.62	0.97
	FleX	0.69	0.82	0.90	0.76	0.83	0.99	0.92	0.86	0.99
	GA-1	0.54	0.77	0.82	0.51	0.54	0.93	0.90	0.88	0.88
	Double	0.73	0.75	0.91	0.70	0.74	0.90	0.90	0.86	0.93
	Mlink	0.67	0.71	0.82	0.70	0.73	0.94	0.91	0.80	0.94
**Average cost**										
RR = 1.5	Scan-c							7.83	15.94	8.49
	Scan-e0							8.77	14.83	9.86
	FleX							8.55	14.20	9.66
	GA-1							10.13	14.99	9.59
	Double							8.81	14.79	11.34
	Mlink							7.60	13.48	12.05
RR = 2.0	Scan-c				6.29	7.01	2.95	5.55	15.41	4.56
	Scan-e0				5.70	8.96	5.76	5.97	13.54	6.04
	FleX				5.87	5.39	4.02	7.40	13.64	5.19
	GA-1				7.93	8.20	3.52	9.18	12.91	5.66
	Double				6.48	6.31	6.57	8.39	12.54	8.84
	Mlink				6.28	6.37	5.36	5.50	11.59	8.04
RR = 3.0	Scan-c	6.95	10.37	5.33	6.43	6.36	0.99	5.00	16.02	3.68
	Scan-e0	3.41	15.06	4.96	2.76	8.95	1.68	4.41	14.36	3.81
	FleX	6.96	8.61	4.53	4.96	4.42	2.05	5.94	14.82	5.62
	GA-1	7.00	7.11	4.76	6.74	8.20	2.77	5.91	13.01	5.81
	Double	6.65	9.23	2.07	5.87	5.75	6.24	6.20	10.55	7.05
	Mlink	6.22	9.71	5.15	5.64	6.49	2.26	3.64	12.95	5.92

Scan-c: circular scan method, *Scan-e0*: standard elliptic scan method, *FleXx*: unrestricted flexible scan method, *GA-1*: strongly penalized genetic algorithm, *Double* and *Mlink*: dynamic minimum spanning tree method with double and maximum link connections, respectively. RR: relative risk in the true cluster.
