# Supplementary material for: Detection of clusters of a rare disease over a large territory: performance of cluster detection methods
Source: Int J Health Geogr. 2011 Oct 4;10:53. doi: 10.1186/1476-072X-10-53 (PMC3204219; doi:10.1186/1476-072X-10-53)
Supplement: Additional file 5 — Study design of published studies on the performance of cluster detection methods. Information on the methods, the study area, the cluster scenarios and the evaluation metrics considered in the published studies. [file 1476-072X-10-53-S5.PDF]

Additional file 5 – Study designs of published studies on the performance of cluster detection methods.

Authors	Methods[1]	Study area		Cluster				No. cases	Simulations: replicates under		Maximum cluster size	α-level	Evaluation metrics
		Country / size	Population	Shape / location	Size (No. units)	Population	Relative risk		H0	H1			
Kulldorff *et al.* 2006 [2]	**Scan-e0**, **Scan-e1**, **Scan-c**	NE United States,<br>245 counties	29.5 million inh.	Circular and elliptic clusters in rural, urban and mixed areas	2, 4, 8, 16	cf. Kulldorff 2003	According to local power	600	99999	10000	n.c.	0.05	usual power
Tango and Takahashi 2005 [3]	**FleX, Scan-c**<br>(+SA as an illustration)	Japan,<br>113 regions	Q1=56704 inh.<br>Median=142320 inh.<br>Q3=200936 inh.	Circular, elliptic and linear	3, 4, 4, 5	n.c.	RR=3.0	200	999	1000	15 units	0.05	- usual power<br>- bivariate power function<br>- average cost
Duczmal *et al.* 2007 [7]	**GA** and SA<br>without and with a penalty	NE United States<br>245 counties	cf Duczmal 2006	11 irregularly shaped<br>(cf. Duczmal 2006)	from 7 to 78 units	n.c.	According to local power	600	100000	10000	8, 12, 20, 30	n.c.	usual power
Assuncao *et al.* 2006 [4]	**Scan-c,** sMST, dMST	SE Brazil<br>291 units	2.2 million inh.	circular, linear, star-shaped, ring-shaped	13, 6, 12, 11	n.c.	According to local power<br>(i.e. ~ 2.5)<br>+ RR=5	420	999<br>for each H1	10000	Scan-c: 20% pop.<br>sMST, dMST: 60 units	0.05	- usual power<br>- no. well-detected areas
Aamodt *et al.* 2006 [11]	**Scan-c,** GAM, BYM	Norway,<br>434 municipalities	4.6 million inh.<br>Q1=2273 inh.<br>Median=4400 inh.<br>Q3=9225 inh.	6 cluster situations<br>(circular, linear, multiple clusters, compact clusters)	14, 6, 15, 70, 345, 125	1.1%, 1.6%, 5.2%, 13%, 89.9%, 32%<br>of the total population	1.2, 1.5, 2.4, 4, 10<br>for each cluster	n.c.<br>(Poisson distributed<br>IR=2.10^{-3})	999<br>for each H1	500	50% pop.	0.05	- usual power<br>- sensibility<br>- specificity<br>- missclassification (no. units)
Costa *et al.* 2005 [12]	**Scan-c,** modified BN	NE United States,<br>245 counties	29.5 million inh.	Circular clusters in rural, urban and mixed areas	1, 2, 4, 8, 16	cf. Kulldorff 2003	According to local power	600	99999	10000	n.c.	0.05	- usual power<br>- detect at least one unit<br>- partial detection
Duczmal *et al.* 2006 [13]	**Scan-c**, **Scan-e0,** SA with a penalty	NE United States,<br>245 counties	29.5 million inh.	linear, U-shaped and ring-shaped	7 to 78	n.c.	According to local power	600	100000	10000	50% no. units	n.c.	usual power
Kulldorff *et al.* 2003 [14]	**Scan-c,** MEET, Bonetti-Pagano	NE United States,<br>245 counties	29.5 million inh.	Circular clusters in rural, urban and mixed areas<br>+ 2 multiple cluster situations	1, 2, 4, 8, 16	E=0.05 to 7.3 (rural)<br>E=14.4 to 34.2 (mixed)<br>E=16 to 155 (urban)	According to local power<br>193 to 3.9 in rural clusters<br>2.9 to 2.1 -- mixed --------<br>2.7 to 1.5 -- urban --------	600 / 6000	100000	10000	50% pop.	0.05<br>0.01	usual power
Song and Kulldorff 2003 [15]	**Scan-c,** BN, CE, MEET, Schwartz, Wittermore, Moran	NE United States,<br>245 counties	29.5 million inh.	Circular clusters in rural, urban and mixed areas	1, 2, 4, 8, 16	E=0.05 to 7.3 (rural)<br>E=14.4 to 34.2 (mixed)<br>E=16 to 155 (urban)	According to local power<br>193 to 3.9 in rural clusters<br>2.9 to 2.1 -- mixed --------<br>2.7 to 1.5 -- urban --------	600 / 6000	99999	10000	n.c.	n.c.	usual power
Takahashi and Tango 2006 [16]	**Scan-c**, **FleX**	Japan,<br>113 regions	n.c.	circular and elliptic clusters<br>(cf. Tango 2005)	3, 4	n.c.	n.c.	200	n.c.	1000	n.c.	0.05	extended power<br>(based on the bivariate power function from Tango 2005)
Tango 2008 [17]	**Scan-c**<br>without and with a restriction	Japan,<br>113 regions	n.c.	circular and elliptic clusters	3, 4, 10, 10	n.c.	3.0 and 2.0<br>or declining with distance (2.5-3; 1.8-2.4)	200 / 45700	10000	1000	50% pop	0.05	bivariate power function (cf. Tango 2005)
Waller *et al.* 2006 [19]	**Scan-c,** Tango's test for clustering	United States<br>259 census tracts	20799 live births	259 circular clusters<br>(centred in turn on each unit)	7	n.c.	RR=3.0	71	1000	1000	50% pop.	0.05	- usual power<br>- detect at least the cluster center
Huang *et al.* 2008 [18]	**Scan-c**, **Scan-e**, **FleX,** CEPP, LISA, ULS	United States<br>3109 counties<br>49 States	n.c.	multiple cluster situations<br>(mostly in urban areas)	167 to 926	Pop=27 to 105 million inh.	1.1 to 2.0	2500, 5000, 10000, 25000 and 50000	10000	1000	50%	0.05	- usual power<br>- sensibility, PPV (with sd and CI)
Costa *et al.* 2011 submitted	**Scan-c**, **Scan-e0**, **Scan-e1**, **Double**, **Mlink,** e-dMST	NE United States,<br>245 counties	29.5 million inh.	Circular clusters in rural, urban and mixed areas<br>+ irregularly shaped<br>(cf. Duczmal 2006)	1, 4, 16<br>(circular)<br>7 to 78<br>(irregular)	E=0.05 to 155 (compact)<br>E=14 to 158 (irregular)	According to local power:<br>1.3 to 193 in circ. clusters<br>1.3 to 2.7 in irreg. clusters<br>+ RR=5.0	600	9999	10000	50% pop<br>(~120 units)	0.05	- usual power<br>- sensibility (pop.)<br>- PPV (pop.)<br>- misclassification (pop.)

[1] BN: Besag and Newell's method; BYM: Hierarchical model developed by Besag, York and Mollie; CE: Cuzick and Edward's method; CEPP: Turnbull et al's cluster evaluation permutation procedure; dMST: dynamic Minimum Spanning Tree method; Double: Double connected spatial method; e-dMST: extended dynamic Minimum Spanning Tree method; FleX: Flexible scan method; GA: Genetic Algorithm method; GAM: Generalized additive model; LISA: Local indicators of spatial association; MEET: Tango's maximized excess events test; Mlink: Maximum linkage spatial method; SA: Simulated annealing method; Scan-c: Circular scan method; Scan-e0: Elliptic scan method with no penalty; Scan-e1: Elliptic scan method with a strong penalty; sMST: static Minimum Spanning Tree method; ULS: Patil and Taillie's Upper Level Set method; MLF: Maxima-likelihood-first algorithm; NGG: non-greedy growth algorithm. bold names correspond to the methods considered in the present study.

SE: southeast; NE: northeast; inh.: inhabitants; Q1: first quartile; Q3: third quartile; E: expected number of cases under the null hypothesis of homogeneous risk; sd: standard deviation; CI: confidence interval No. units: number of units included in the study; n.c.: information not communicated by the authors
